# Videothoracoscopic management of hemoptysis due to anomalous bronchial vessel treated with multiple bronchial artery embolizations: a case report

**DOI:** 10.3389/fped.2024.1431590

**Published:** 2024-11-19

**Authors:** Alice Gismondi, Simone Frediani, Valerio Pardi, Ivan Pietro Aloi, Arianna Bertocchini, Antonella Accinni, Alessandro Inserra

**Affiliations:** General and Thoracic Pediatric Surgery Unit, Bambino Gesù Children’s Hospital, IRCCS, Rome, Italy

**Keywords:** videothoracoscopic clipping, BAE, anomalous vessel, hemoptysis, children

## Abstract

**Introduction:**

Hemoptysis is an alarming clinical presentation caused by a vast number of primitive conditions (infectious, malignancies, malformations, vasculitis). However, at the root of hemoptysis, there is always a “noxa patogena” altering vessel structure, usually bronchial arteries, which are characterized by high pressure. Bronchial artery embolization (BAE) is the first-line treatment for hemoptysis for its technical and clinical success, although the long-term overall outcome is not equally adequate.

**Case report:**

A 12-year-old boy was referred to our hospital for massive hemoptysis after a history of recurrent episodes since the age of 3. The patient had been diagnosed with bilateral and widespread bronchial artery hypertrophy at another hospital and treated with several BAE procedures. We performed BAE to stabilize the child as well as an angio-CT scan, which confirmed the presence of the recently placed coil to embolize a hypertrophic bronchial arteriosus branch originating from the left thyrocervical trunk and directed to the right lower lobe. Results of previous embolization (metal coils) were found at the origin of the right inferior thyroid artery and the right costo-cervical trunk. After 21 months since his first admission to our hospital, the patient was transferred by air ambulance for a massive hemoptysis recurrence. Further BAE of the previously coiled vessel coming from the right succlavia (and right inferior thyroid artery) was impossible to perform due to the presence of the coils positioned in the past. A thoracoscopic approach was chosen: the previously identified anomalous vessel was isolated and ligated using double metal clips, two on both the proximal and distal sides. Accurate exploration of the thoracic cavity was accomplished, verifying the absence of collateral vessels coming from the diaphragmatic side. The patient was discharged in four days in good clinical.

**Discussion:**

Although bare-minimum invasive embolism (BAE) is still the gold standard for treatment, there are situations when it may not produce the desired clinical outcome and increase the risk of rebleeding. In these situations, minimally invasive surgical procedures using a videothoracoscopic approach can be beneficial if there is a suspicion of an aberrant vessel on a DSA or CT scan.

## Introduction

Hemoptysis is defined as the expectoration of blood from the alveoli or airways of the lower respiratory tract. Literature commonly defines hemoptysis as massive when it causes clinical consequences such as respiratory failure from airway obstruction or hemodynamic instability (1, 2).

Massive hemoptysis is a life-threatening condition that requires immediate treatment. The causes of hemoptysis are heterogeneous, ranging from bronchitis, pneumonia, tuberculosis, cystic fibrosis, malignancies, vascular and collagen diseases (such as Goodpasture's syndrome, Behçet's disease, systemic lupus erythematosus, granulomatosis with polyangiitis, Henoch-Schonlein purpura, and rheumatoid arthritis), drugs and narcotics, pulmonary embolism, and pulmonary hypertension. However, at the root of hemoptysis, there is always a “noxa patogena” altering vessel structure, usually bronchial arteries, which are characterized by high pressure.

Bronchial artery embolization (BAE) is the first-line treatment for hemoptysis for its technical and clinical success (greater than 73.7% and 84.2%, respectively) (3). However, Angio-CT scan must be attempted as a first diagnostic step in approaching hemoptysis in order to identify in advance the artery concerned.

## Case report

A 12-year-old was referred to our hospital for recurrent episodes of hemoptysis since the age of 3, several of which required BAE with the placement of metal coils. The diagnosis of bilateral and widespread bronchial artery hypertrophy had been made at another hospital. The parents reported that genetic testing or other in-depth investigations had never been performed. However, they were unable to provide documentation from the other hospital.

**Figure 1:**
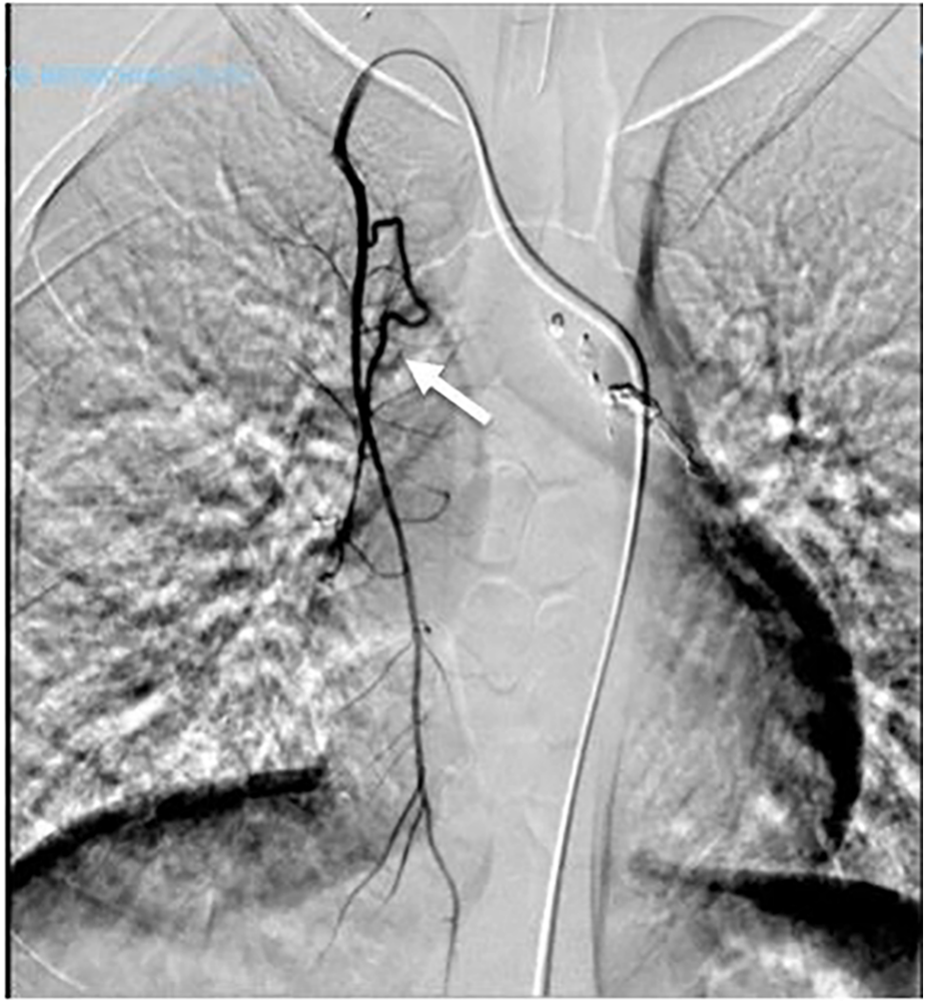
Bronchial arterography (the arrow indicates the anomalous vessel).

The patient was admitted to our emergency department for massive hemoptysis, treated with BAE, and supported with blood transfusions. After stabilizing the patient, we transferred him to our department and prescribed a CT bronchial angiography scan. The exam showed the results of the recent embolization of a hypertrophic bronchial arteriosus branch originating from the left thyrocervical trunk and directed to the right lower lobe, with evidence of hyperdense material along the course of the anomalous vessel at a para-tracheal and peri-bronchial level (right main bronchus, intermediate-lower bronchus, and posterior segmentary branching). Results of previous embolization (metal coils) were found at the origin of the right inferior thyroid artery and the right costo-cervical trunk. Another bronchial arteriosus branch directed to the right lower lobe was reported in the ilo-mediastinal area, presumably originating in the right inferior thyroid artery (immediately downstream from the metal coil previously mentioned), and its course is aberrant and right paramedian-prevertebral. Almost complete consolidation of the right lower lobe, obliteration of afferent bronchial branches, and multiple ground glass areas (related to anamnestic history) were also reported (Figure 1).

An endoscopic exam and airway toilette were performed with clinical improvement. Spirometry, bubble- tests, EKG, echocardiography, brain RMN, and microbiological tests were carried out, all of which resulted within range.

The case was discussed at a multidisciplinary meeting, and the patient was discharged with a follow-up program, including monthly day hospital and genetic testing for Rendu-Osler-Weber syndrome, which resulted in negative results for pathogenetic variants.

The patient was readmitted to our emergency department 14 months later due to a reported hemoptysis, which the ER personnel did not witness. A chest radiograph was performed with a negative outcome. We discharged the patient in good clinical condition.

21 months after his first admission to our hospital, the patient was transferred by air ambulance due to a massive hemoptysis recurrence. Tranexamic acid was beneficially administered. An angio-CT scan was performed, and there was no evidence of active bleeding. However, widespread endoalveolar hemorrhagic infarction was visible in the right lower lobe and in the medium-lobe lateral segment. A varix-like ectasia of a pulmonary arterial angle directed to the right lower lobe was shown, most likely replenished by a collateral bronchial vessel. The main angle is believed to come from the distal portion of the previously coiled vessel coming from the right succlavia (and right inferior thyroid artery).

A bronchial artery embolization was attempted without success, due to the presence of the previously inserted coils which prevented the advancement of the instrument. The only exception was a minor collateral, the coiled internal medial mammary artery.

We discussed the case again and decided on a surgical approach to try to remove the vascular abnormality permanently. Two options were presented: videothoracoscopic approach with vessel ligation or lobectomy if the first option were to be deemed unfeasible.

**Figure 2:**
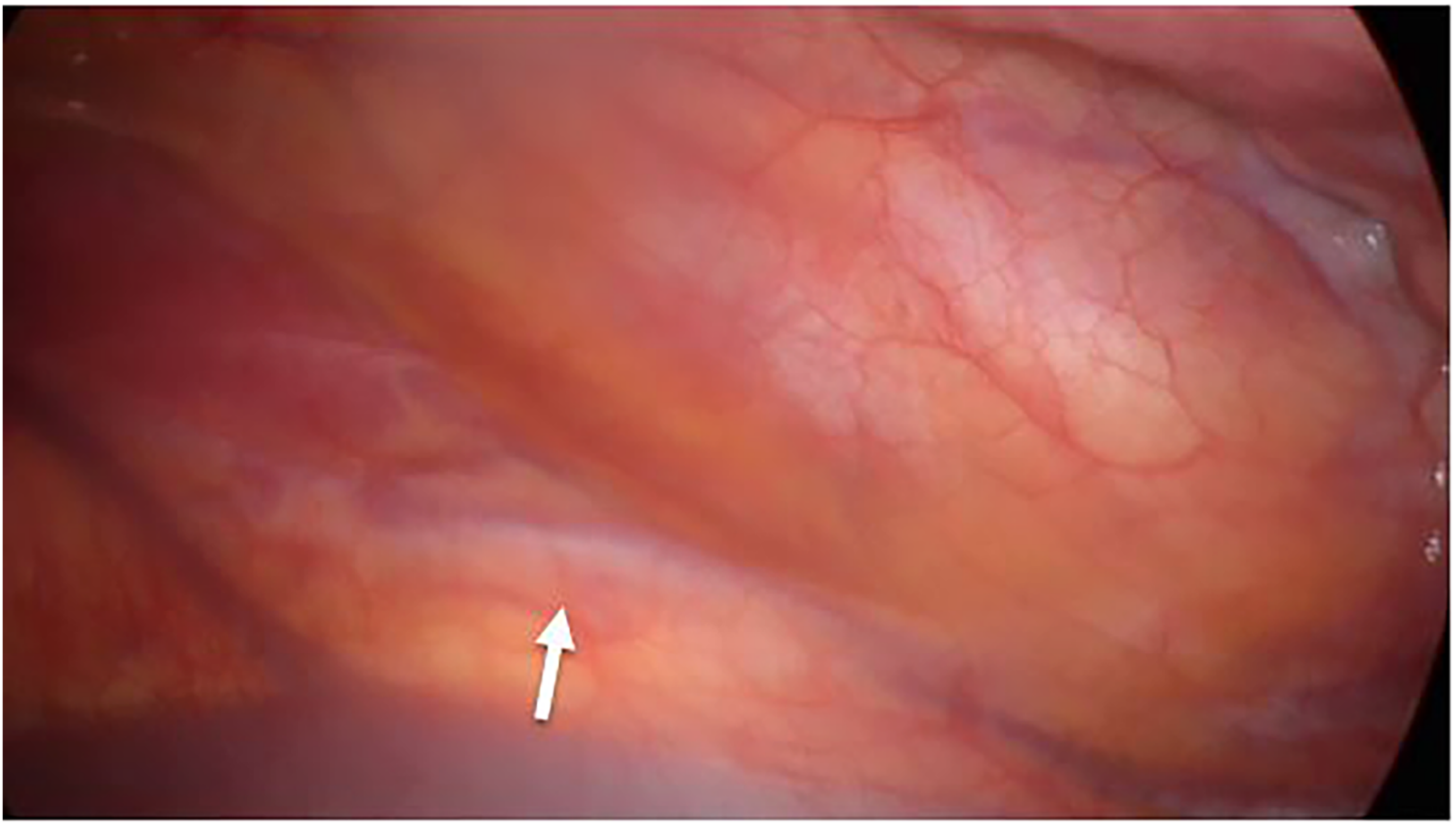
Videothoracoscopic images (VATS). The arrow indicates the anomalous vessel.

**Figure 3:**
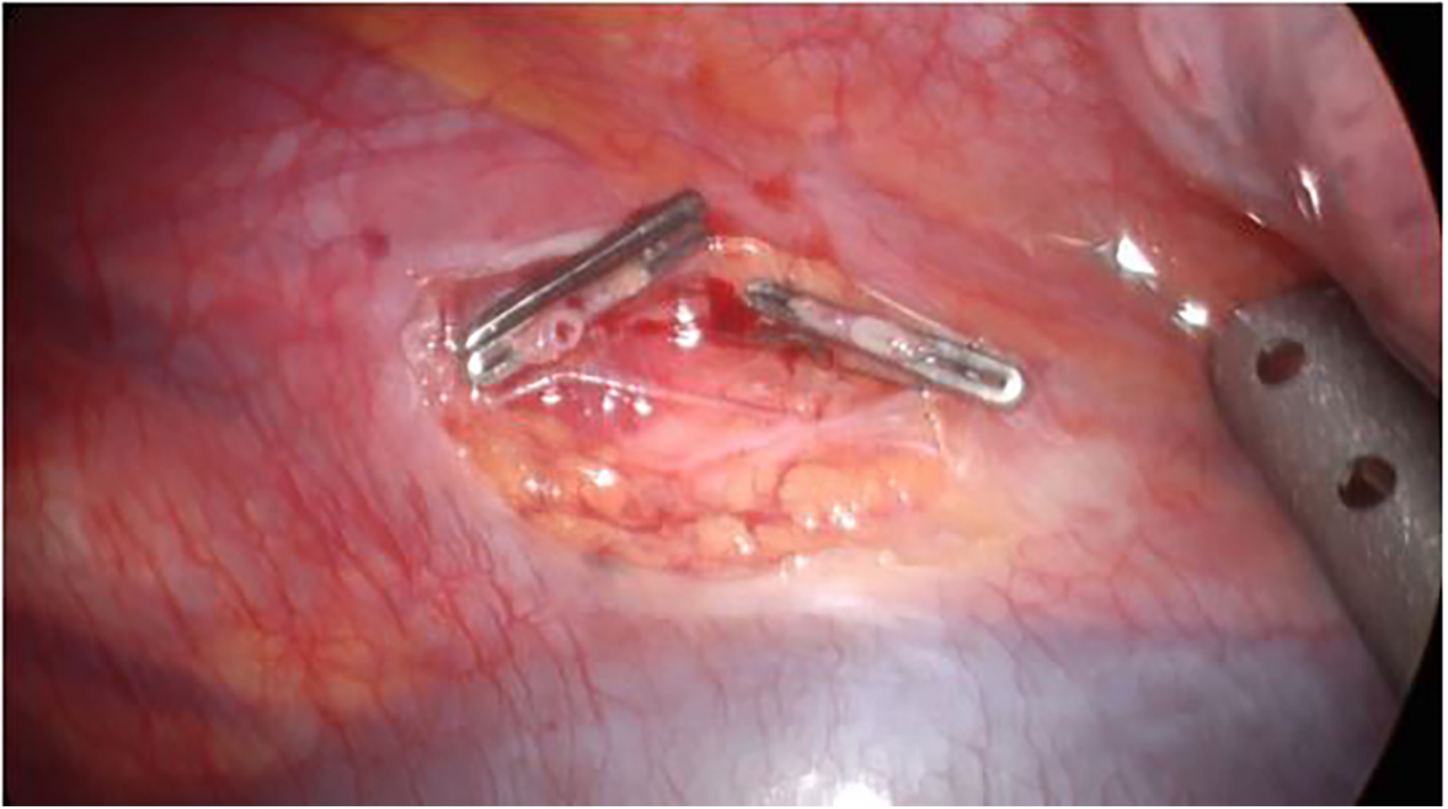
Videothoracoscopic images (VATS). Anomalous vessel was isolated and ligated using double metal clips.

The first trocar was positioned at the level of the lower edge of the scapular angle; after right lung exclusion, the azygos vein and superior afferent vessel were identified. We identified the aberrant vessel medially, underneath the pleura (Figure 2). Two more operative trocars were positioned in the III and V intercostal spaces along the posterior axillary line. A pleural incision was performed, and the previously identified anomalous vessel was isolated and ligated using double metal clips, a couple on both the proximal and distal sides (Figure 3). Accurate exploration of the thoracic cavity was accomplished, verifying the absence of collateral vessels coming from the diaphragmatic side. A thoracic drainage system was left in place using a Thopax Medela aspiration device.

The thoracic drainage was removed on the fourth post-operative day, and the patient was discharged in good clinical condition.

The follow-up was exclusively from a clinical point of view. given that the patient was asymptomatic for more than a year, for reasons of radiation protection we did not consider it necessary to carry out new radiographic checks.

## Discussion

Hemoptysis, especially in the form of massive hemoptysis, is a clinical phenomenon that requires immediate intervention, stabilizing the patient being paramount prior to discerning the causes. Indeed, the conditions provoking hemoptysis are extremely heterogeneous, considering the age of the patient, the environmental background, and other factors (4). Bronchial artery embolization (BAE) is a life-saving treatment for massive hemoptysis (5), as bronchial arteries are responsible in 90% of the cases (pulmonary arteries represent 5% of the cases; the rest are caused by the aorta or other systemic arteries) (1). However, an evaluation of therapeutic outcomes, particularly the difference between immediate effectiveness and long-term results, must be carried out. Immediate effectiveness is defined by technical success and immediate control of bleeding, whereas long-term success is characterized by the absence of a recurrence of bleeding after a successful BAE (6).

In our case report, although we can affirm that the BAE performed was technically successful, the overall outcome was not, as the coils positioned previously were an obstacle to the correct execution of the procedure meaning surgery was required.

According to a meta-analysis carried out by Karlafti et al. (3), the recurrence rate after BAE is 21.46%. The causes of recurrence are numerous and time-related: the development or persistence of collateral circulation, the new development of metastasis, cystic fibrosis, and lung mycosis. There is no doubt that embolization should be a first-line treatment, especially in an emergency setting where timing is paramount, and the main goal is to ensure hemodynamic stability and airway clearing. Once the patient stabilizes properly, we must conduct a thorough investigation to determine the underlying causes, if not already known (7).

We believe that surgical treatment is advisable, especially when multiple BAEs have been unsuccessfully performed and the underlying cause of hemoptysis is a primary condition, such as vascular malformations and aberrant vessels. Yeh YT et al. (8). reported a case of bronchial Dieulafoy's disease in a pediatric patient undergoing surgery, considering the likelihood of recurrence with exclusive embolization. However, they emphasized the significance of arteriography before surgery, advocating for a collaborative approach to recurrent hemoptysis resulting from vascular abnormalities. Videothoracoscopic clipping of the anomalous vessel proved to be the best treatment in the cases described by Kentaroh et al. (9) and the Okur study group (10), where the cause of hemoptysis was an aneurysmatic arteriovenous malformation in a young adult female in the first article and an abnormal arterial branch originating from the right internal mammary artery in a 53-year-old woman in the latter case report.

Although in the literature there are few reports of pediatric patients treated with surgery, we believe that, with a prior and thorough arteriographic study, mini-invasive surgery should be considered as a valid alternative for first-line treatment in carrier patients of vascular abnormalities, given the high probability of failure of the BAE and of hemoptysis recurrence. Supplying vessels, coil migration, or technical issues are the most frequent causes of embolization failure. BAE is undoubtedly the treatment of choice in emergencies where the stabilization of the patient through the control of bleeding is paramount. However, once the underlying cause has been assessed, videothoracoscopic surgery can provide a long-term successful outcome, requiring only one general anesthesia and hospital stay compared to BAE. In our case we chose vessel ligation technique; however, if the cause of hemoptysis resides in primitive or secondary lung parenchyma damage, lobectomy or wedge resection may represent the only solution as reported in literature. McNamee et al. describe a case of a young adult woman affected by Congenital Lobar Emphysema who developed massive hemoptysis and required urgent videthoracoscopic (VATS) resection of her right lung upper lobe (11). In a retrospective review of pulmonary arteriovenous malformation Fish et al. concluded that surgical resection may ultimately be required in refractory and multifocal disease, as there is a high recurrence rate after BAE due to underlying lung parenchyma damage (12).

In our limited experience, we believe that a mini-invasive surgical procedure should be considered as a treatment of choice when risk factors for hemoptysis recurrence are present from the beginning (non-actively bleeding vascular malformation, primitive lung disease) or there are factors suggesting recurrence (history of multiple BAEs, thus multiple coils). However, further studies and case analyses should be performed to provide a definitive guideline for hemoptysis thoracoscopic therapy.

## Data Availability

The original contributions presented in the study are included in the article/Supplementary Material, further inquiries can be directed to the corresponding author.
